# Chaotic Image Encryption Based on Running-Key Related to Plaintext

**DOI:** 10.1155/2014/490179

**Published:** 2014-02-23

**Authors:** Cao Guanghui, Hu Kai, Zhang Yizhi, Zhou Jun, Zhang Xing

**Affiliations:** ^1^Electronic & Information Engineering College, Liaoning University of Technology, Jinzhou 121001, China; ^2^School of Computer Science and Technology, Beihang University, Beijing 100191, China

## Abstract

In the field of chaotic image encryption, the algorithm based on correlating key with plaintext has become a new developing direction. However, for this kind of algorithm, some shortcomings in resistance to reconstruction attack, efficient utilization of chaotic resource, and reducing dynamical degradation of digital chaos are found. In order to solve these problems and further enhance the security of encryption algorithm, based on disturbance and feedback mechanism, we present a new image encryption scheme. In the running-key generation stage, by successively disturbing chaotic stream with cipher-text, the relation of running-key to plaintext is established, reconstruction attack is avoided, effective use of chaotic resource is guaranteed, and dynamical degradation of digital chaos is minimized. In the image encryption stage, by introducing random-feedback mechanism, the difficulty of breaking this scheme is increased. Comparing with the-state-of-the-art algorithms, our scheme exhibits good properties such as large key space, long key period, and extreme sensitivity to the initial key and plaintext. Therefore, it can resist brute-force, reconstruction attack, and differential attack.

## 1. Introduction

With the rapid development of network and multimedia technology, a large number of images are transmitted and stored in the network. Interactivity, openness, and sharing of network as well as easiness of copying and modifying a digital image, while providing the convenience for users, also provide the opportunity for attackers to steal or tamper secret data [1]. For instance, in June 2013, Edward Snowden leaks the PRISM documents to the media. The major content is that, since 2007, the National Security Agency collects documents, connection logs, photos, videos, emails, and more to acquire personal contact information and action. In addition, according to the report a survey of China Internet Network Security Situation, published by China Computer Emergency Response Team (2012), more than 141.97 millions of computers in the territory of China are controlled by about 0.73 millions of abroad Trojan horses or bonnets. Attackers can acquire any information in these controlled hosts. These facts show that the secret images, whether transmitted through open network or stored in insecure station, need a maximum protection. With the advent of cloud computing and the arrival of big data era, the demand for security becomes extremely high. Among several protection methods in the literature, encryption is one of the most efficient and common methods. However, the inherent characteristics of image, such as high redundancy of data, strong correlation among adjacent pixels, and less sensitivity to data change and its structure property make traditional document encryption schemes unsuitable for image encryption [1, 2]. In recent years, with further study in chaos theory, many desirable encryption characteristics of chaotic sequence, for example, nonperiodicity, like-random behavior, sensitivity, and easy to implement, have been found. These features help to enhance the security and reduce the cost of implementation for encryption algorithm. Thus, image encryption based on chaos has become one of the most important image protection methods.

## 2. Related Work

In 1998, Fridrich first proposed a representative chaotic image encryption scheme, which has confusion-diffusion integration mechanism [3]. In order to enhance the role of chaos in this kind of integration mechanism, Chen et al. [4], based on 3D cat map, proposed an image encryption scheme. Afterwards, three other schemes were presented in [5–7]. Essentially, these schemes have the same encryption principle; that is, confusion and diffusion perform alternatively. However, paper [8] (2008) analyzes this kind of scheme and finds some problem such as weak sensitivity to the change of plain-images or key stream and the flaw of diffusion function and pseudorandom sequence. Obviously, these shortcomings violate the security rules proposed in [9]. On top of that, paper [10] find another defect of Fridrich integration mechanism; that is, neither initial-key nor running-key of an encryption system is related to plaintext. So, if using the same user keys, encrypt scheme would use the same initial-key and running-key to encrypt different images. Practically, user keys do not frequently change. Consequently, this kind of encryption algorithm is cracked. Under these circumstances, in order to design more secure image encryption, studying image encryption in which initial-key or running-key is related not only to user keys but also to plaintext has received increased attention in the research community.

According to the type of key related to plaintext, the existing image encryption algorithms can be divided into two categories. One is based on initial-key related to plaintext. The other is based on running-key related to plaintext.

(*1) Image Encryption Based on Initial-Key Related to Plaintext.* Paper [11] proposed chaotic image encryption by correlating initial key with plaintext. In the initial-key generation stage, it uses plaintext to disturb the current chaotic number and then takes the disturbed chaotic number as the next input of chaotic iteration. Repeat this process and use the last output value as the initial key. By doing so, the correlation between initial-key and plaintext is created. Paper [12] proposed remote-sensing image encryption in hybrid domains, which first gets the hash value of original image and then calculates the initial key of the encryption system through eight formulae. These formulae use hash value as parameters. Thus, the algorithm realizes the correlation between initial key and plaintext. After that, by using the same method as paper [12], paper [13] established the correlation between initial key and plaintext. Paper [1] proposed a color image encryption algorithm based on DNA sequence operation and hyperchaotic system, which first encodes the image with DNA encoding rules and then gets three Hamming distances through image channels, next, transforms them into three decimal numbers, after that, adds them to initial values of chaotic system, finally, gets initial key. Through this process, the correlation is established.

(*2) Image Encryption Based on Running-Key Related to Plaintext. *Paper [14] proposed a new image encryption algorithm based on 3D chaotic map. This algorithm does not use the whole chaotic numbers, but only utilizes partial elements as running key. In chaotic sequence, those used elements keep certain interval, which is determined by the current cipher-text. Due to cipher-text closely related to plaintext, running-key is indirectly related with plaintext.

For the first category, although these algorithms all establish the correlation between the initial key and plaintext, thus being capable of resisting attack method proposed by Solak et al. [10]. Yet chaotic stream, which is used to generate running-key, is totally determined by the initial key and thus its orbit does not dynamically change in the encryption process, which makes encryption algorithm vulnerable to reconstruction attack. For the second one, although algorithm is of good security, yet it only uses a part of the chaotic stream when generating running-key and wastes each element generated but not used. Therefore, it not only fails to not only fails to make full use of chaos resources, but also wastes the expensive time of the system. In addition, the common defect of these two categories of encryption schemes is that they do not consider digital chaotic dynamical degradation. Aiming at the above limitations, an efficient and secure image encryption scheme based on cipher-text disturbing chaotic stream is proposed.

## 3. Image Encryption Based on Running-Key Related to Plaintext

### 3.1. Notation Definitions

*f*: A procedure or function that transforms any chaotic sequence from any distribution into (0,1) uniform distribution. For example: A universal algorithm for transforming chaotic sequence into uniform pseudorandom sequence [15], or shift-and-cut method [16] as follows: *f*(*x*) = |10^*k*^ *x* − fix(10^*k*^ *x*)|, here *x* refers to chaotic number.
*g*: Any function that can transform uniform (0,1) random variable into uniform integer space. A concrete example is *g*(*u*) = int⁡(*vu*) + 1, *v* ∈ *ℤ*, which can map *u* ∈ *U*(0,1) into *g*(*u*) ∈ *U*[1, *v*], where *U*[*a*, *b*] represents uniform distribution over [*a*, *b*]; int⁡(*x*) is a function that truncates the decimal portion and remains the integer portion of the value *x*.
*δ*: Any function that can map an integer number from (0,255) to (0,1). A function like this: *δ*(*s*, *y*) =  int⁡((10^*s*^∗*y*)/257)∗10^−*s*^, *y* ∈ (0,255) ⊂ *Z*, which can map *y* to (0,1) with *s* significant digits.
*h*: A disturbing function implementing the interaction between current chaotic output and the value of *δ* function. Math expression is *x*′ = *h*(*x*, *δ*(*n*, cipher)). The simplest *h* function is *h*(*x*, *δ*) = (*x* + *δ*(*n*, cipher))mod⁡1.

### 3.2. Basic Idea

**Figure 1:**
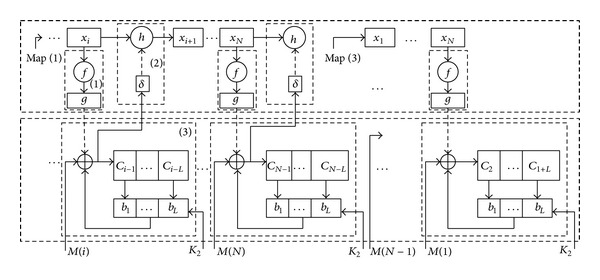
Block diagram of our scheme.

Our scheme (see Figure 1) is comprised of three parts. The first part is key uniformity module, which includes chaotic sequence uniformity function *f* and space transformation function *g*, implementing transformation from any chaotic sequence to cipher-key. The second is chaotic sequence disturbing module, by disturbing function *h*, achieving the function of reducing dynamical degradation of digital chaos and generating one time running-key stream. The last is random feedback encryption module, in which both length and position of feedback cipher-text are random, increasing the difficulty of breaking our scheme.

### 3.3. Algorithm Implementation

**Figure 2:**
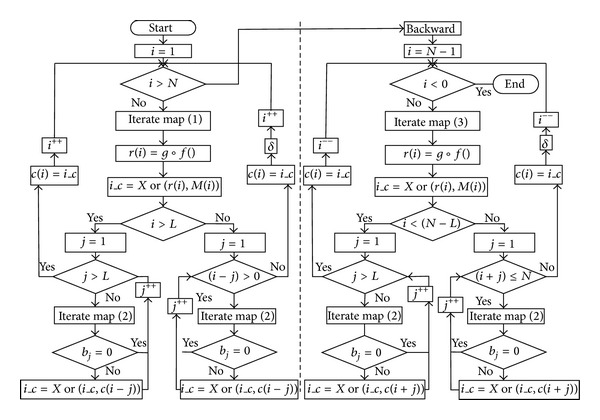
Encryption flowchart of our scheme.

The encryption processes, which include the forward and backward encryption, are shown in Figure 2. The specific procedure is described in detail as follows.

#### 3.3.1. Encryption Process

*Initialization.* Select three chaotic maps. The first one, map (1), is used to forward encryption. The second one, map (2), is used to generate a binary vector. The last one, map (3), is used to backward encryption. Set user key, which includes the initial values and parameters of chaotic maps, and related function parameters. *N* is assigned to the number of image pixels.

**Phase 1 (Forward Encryption)**

(1) Generate a chaotic number *x* by iterating map (1). Next, get (0,1) pseudorandom number by applying function *f* to *x* and then obtain the current running key *r*(*i*) through function *g*;

(2) generate a binary random vector *V* = (*b*_1_, *b*_2_,…, *b*_*L*_) by iterating map (2);

(3) encrypt the current plain-pixel, formulae as follows:

(1) c(i)=(r(i)⊕M(i)⊕b1∗c(i−1)⊕b2∗c(i−2)⊕⋯⊕bi−1∗c(1)), i≤L,c(i)=(r(i)⊕M(i)⊕b1∗c(i−1)⊕b2∗c(i−2)⊕⋯⊕bL∗c(i−L)), i>L;

(4) map the current cipher-pixel into (0,1) real number with *δ* function. Next, use it to disturb the current output of map (1) and then take the disturbed result as the next input of map (1);

(5) repeat steps (1)–(4) until the last pixel of the image.

In order to guarantee plaintext sensitivity in any position, we take the above cipher image as an intermediate image; start with the (*N*−1)th pixel and reversely encrypt the image.

**Phase 2 (Backward Encryption)**

(1) Generate a chaotic number by iterating map (3). Next, apply uniformity function *f* to it to get the (0,1) pseudorandom number and then obtain the current running key *r*(*i*) through function *g*;

(2) generate a binary random vector *V* = (*b*_1_, *b*_2_,…, *b*_*L*_) by iterating map (2);

(3) encrypt the current pixel, formulae as follows:

(2) c(i)=(r(i)⊕M(i)⊕b1∗c(i+1)⊕b2∗c(i+2)⊕⋯⊕bj∗c(i+j)), (i+j)≤N,c(i)=(r(i)⊕M(i)⊕b1∗c(i+1)⊕b2∗c(i+2)⊕⋯⊕bL∗c(i+L)), i<N−L;

(4) map the current cipher-pixel into (0,1) real number with *δ* function. Next, use it to disturb the current output of map (3) and then take the disturbed result as the input of map (3);

(5) repeat steps (1)–(4) until the first pixel of the image.

#### 3.3.2. Decryption Process

The decryption process is very similar to the encryption process except that first decrypt cipher-text is generated in the backward encryption process and then do it in the forward encryption process.

## 4. Encryption Results

In order to verify practicality and efficiency of our scheme, a lot of encryption experiments have been done. Here we illustrate a simplest example. Three chaotic maps all take Logistic equation *x* = *μ*∗*x*∗(1 − *x*). Their differences lie in initial values and control parameters, here *μ*_1_ = 3.8898, *μ*_2_ = 3.98, *μ*_3_ = 3.96, *x*_1_ = 0.01, *x*_2_ = 0.3, and *x*_3_ = 0.3, where *μ*_*i*_ and *x*_*i*_ refer to the parameter and the initial value of map *i*, respectively. Additionally, *L* = 4 and *m* = *n* = 10.

**Figure 3:**
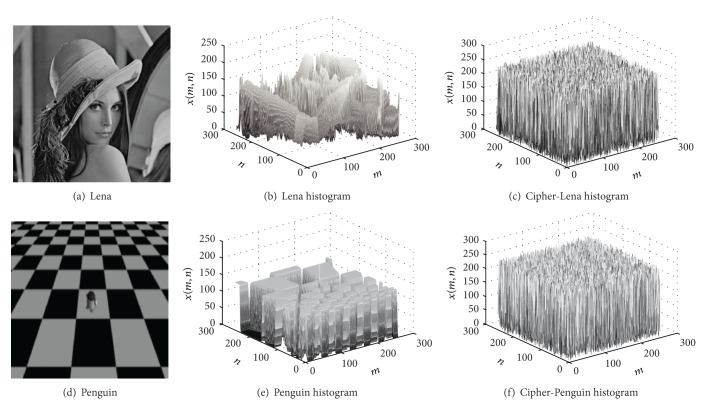
Encryption results analyses.

Take Lena image and Penguin image as examples, the space histograms for the original images and the corresponding cipher-images are showed in Figure 3. From these results, we can see that our scheme has good cipher-text space distribution.

## 5. Performance and Security Analysis

### 5.1. Key Space

Our scheme includes the following user keys: initial values and control parameters of chaotic systems, feedback length *L*, parameter(s) of uniformity function *f*, and parameter *u* of function *g*. Even if we consider the simplest case: three chaotic maps all take Logistic map; only take their initial values and parameters as key space (suppose the greatest accuracy of variables is 10^−14^), while omitting other key possible values. The key space is still at least *O*(10^64^), which is large enough to resist all kinds of brute-force attack.

### 5.2. Dynamical Degradation Analysis of Digital Chaos

At present, image encryption schemes based on the correlation between cipher-key and plaintext have dynamical degradation problem. Paper [17] gives three practical solutions as possible remedies: using higher precision, cascading multiple chaotic systems, and (pseudo-)randomly disturbing chaotic system. Furthermore, it points out that disturbance-based solution is superior to others. Thus, our scheme utilizes cipher-text disturbing chaotic stream method to minimize the impact of chaotic degradation on encryption system security.

**Figure 4:**
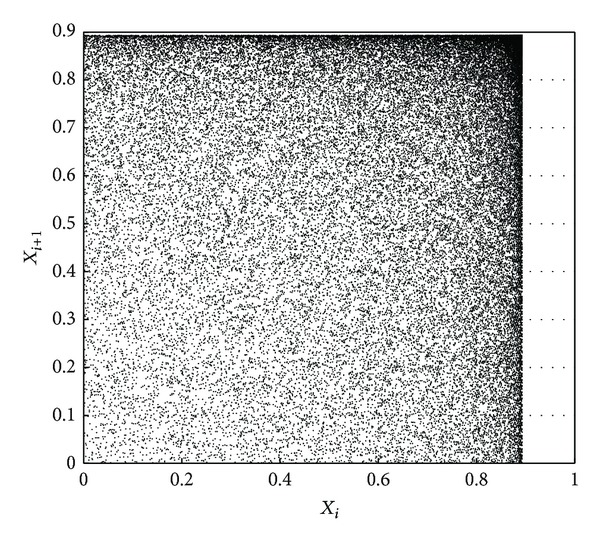
Nearly full space trajectory.

**Figure 5:**
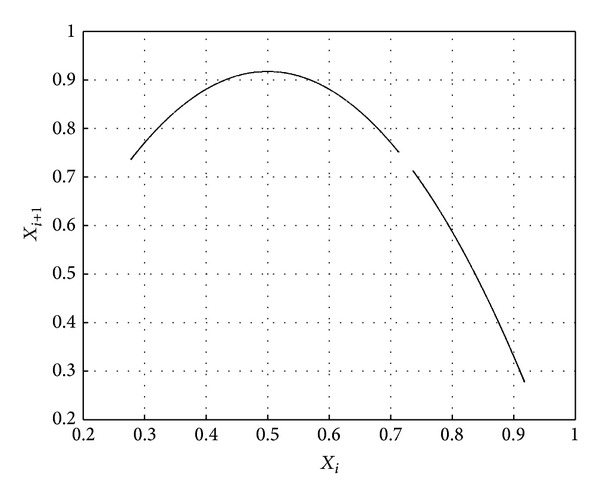
Single trajectory.

**Figure 6:**
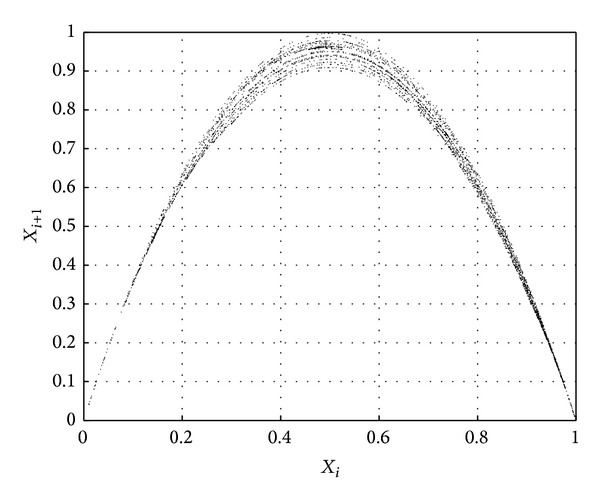
Compound trajectory.

In order to verify the randomness and periodicity of chaotic sequences generated by our scheme, the phase space (*x*_*i*_, *x*_*i*+1_) generated by using our scheme is shown in Figure 4. For comparison, phase spaces generated by using logistic map and method of literature [18] are also shown in Figures 5 and 6, respectively.

One can see that the phase space in Figure 5 is the single trajectory. On top of that, it has many breakpoints. These facts show that such chaotic sequence has strong correlation and short length of period. When compared with Figure 5, the trajectory of phase space in Figure 6 is compound trajectory, which shows that the sequence's correlation becomes weak and its period becomes long. It should be noted that in Figure 4, nearly full space trajectory is generated, not limited by fixed orbits. Thus, randomness and periodicity of chaotic sequence are superior to the other two. These experiments show that our scheme can minimize dynamical degradation of digital chaos.

### 5.3. Reconstruction Attack Analysis

Image encryption scheme, which uses chaotic sequence to generate cipher key, may be attacked by phase space reconstruction. The theory foundation of such attack is Takens' delay embedding theorem [19]. But in order to use this theory, acquiring a sequence of observations of the state of a dynamical system is the precondition. In our scheme, The chaotic sequence observed by any attacker has been disturbed by cipher-text. According to chaotic sensitivity, the disturbed sequence is totally different from the original chaotic sequence. Thus, phase space reconstruction attack is impossible.

### 5.4. One-Time Running Key Stream

**Figure 7:**
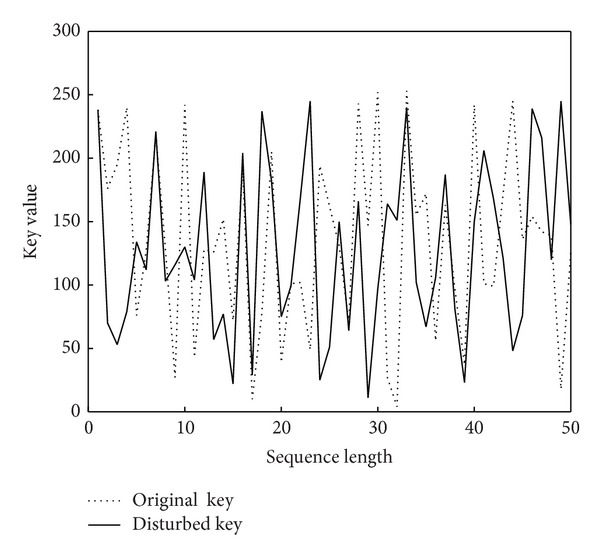
Running keys before and after disturbance.

**Figure 8:**
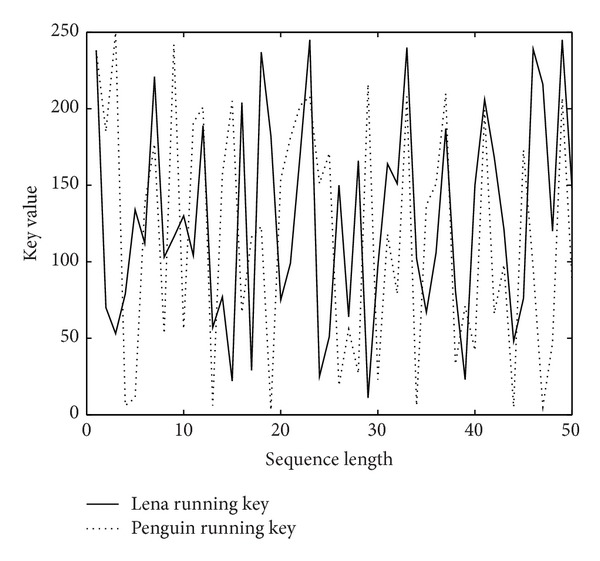
Two different running keys.

According to Shannon's theory [20], perfect secret system needs one time pad. In our scheme, under the condition of the same user key, Figure 7 shows the running keys generated by chaotic sequence before and after disturbance. In which the dotted line represents undisturbed running key, the solid line represents disturbed running key used when system encrypts Lena image (see Figure 3(a)).  Figure 8 shows two running keys, represented by a dotted line and a solid line, used when system encrypts Lena image and Penguin image (see Figure 3(d)), respectively.

As can be seen from these results, key generation mechanism in our scheme can guarantee one time running key stream.

### 5.5. Adjacent Pixel Correlation

Correlation test uses formula (3).

(3) Cr=(N∑j=1Nxj×yj−∑j=1Nxj×∑j=1Nyj)×((N∑j=1Nxj2−(∑j=1Nxj)2)  ×(N∑j=1Nyj2−(∑j=1Nyj)2))−1,

where *x*_*j*_ and *y*_*j*_ represent adjacent pixel values, *N* represents image size. For a meaningful image, adjacent pixels usually have a relatively large correlation. For a cipher image, pixel correlation should be as small as possible. For comparison, Rand image is introduced, which is composed of random elements. Seen from Table 1, adjacent pixels in cipher image have very small correlation, which proves our scheme's effectiveness.

### 5.6. Sensitivity Analysis

A good encryption algorithm should be sensitive to the changes of plaintext or key.

#### 5.6.1. Plaintext Sensitivity Analysis

Attacker often makes a tiny change in plain image and then observes the changes of corresponding cipher image. If the change is small, the attacker can find out the relation between plain image and cipher image and hence breaks the encryption process. Conversely, differential analysis becomes not significant [13]. Plaintext sensitivity can be investigated through pixel change ratio and bit change ratio. The former can be measured by NPCR (number of pixel change rate) and UACI (unified average change intensity) [4]. The latter can be examined by avalanche effect.

Table 2 shows the results of plain-image sensitivity analysis. The NPCR is over 99%, UACI is over 33%, and bit flip ratio is close to 50%. These results show that our scheme is extremely sensitive to plain-image, therefore, can effectively resist chosen-plaintext attack.

#### 5.6.2. User Key Sensitivity Analysis

User key refers to some values given by a user used to set the parameters of an encryption scheme. The sensitivity of cipher-image to user key can be analyzed from two aspects. One is correlation. The other is decryption ability.

Correlation refers to the relationship between two cipher-images. Its strength can be measured by correlation coefficients. Here, two cipher images are generated with the same plain-image, slightly different user keys. According to the definition of user key sensitivity, if cipher image sensitively depends on the user key, encrypting the same image with slightly different user keys would generate two completely different cipher images, which have little correlation. Formula (3) is adopted to calculate correlation coefficients, where *x*_*j*_, *y*_*j*_ represent pixel values of two cipher images, respectively. Experiment is as follows.

Suppose primary user key is *r*_1_ = 3.8898, *r*_2_ = 3.98, *r*_3_ = 3.96, *x*_1_ = 0.01, *x*_2_ = 0.3, and *x*_3_ = 0.3. Each time, we slightly alter a component of the primary key to obtain a new user key. For instance, if altering the 3rd component, we can get the new user key: *r*_1_ = 3.8898, *r*_2_ = 3.98, *r*_3_ = 3.96 + Δ, *x*_1_ = 0.01, *x*_2_ = 0.3, and *x*_3_ = 0.3. Calculate the correlation coefficient of two cipher images, which are encrypted with the primary key and the new user key. Seen from Table 3, two cipher images have little correlation, which shows that our scheme is sensitive to user key.

**Figure 9:**
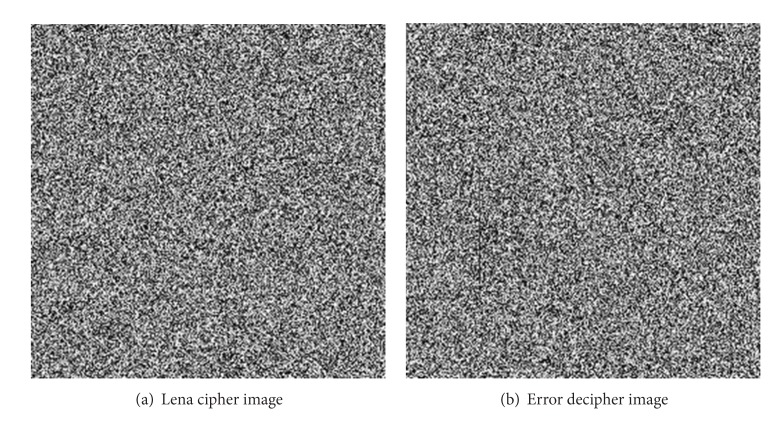
Decryption sensitive analysis.

In the decryption ability, Figure 9(a) shows the Lena-cipher image encrypted with the primary user key.  Figure 9(b) shows decrypted image of Figure 9(a) with the user key generated by making a Δ*r*_1_ alteration to the primary user key.

As we have expected, if the component, no matter what, of the primary user key is changed slightly, the decrypted image would be completely different from the original image. Thus, our algorithm is extremely sensitive to the change of user key.

### 5.7. Min-Entropy Analysis

In chaotic image encryption field, Shannon's entropy is always utilized to do statistics analysis. But Shannon's entropy measures the amount of randomness that a distribution contains on average. Considering the practical methods of attack, we think min-entropy [21] is more suitable to do that.

Min-entropy: *H*_*∞*_(*x*) = min_*x*∈{0,1}^*n*^_log⁡1/pr[*X* = *x*], *X* ∈ {0, 1}^*n*^. Equivalently, a distribution has min-entropy at least *k* if the probability of each element is bounded by 2^−*k*^. Intuitively, such a distribution contains *k* random bits. Min-entropy measures the amount of randomness on the worst case. In order to support our opinion, both Shannon-entropy and min-entropy are all shown in Table 4.

From Table 4, we can see that Shannon entropy of cipher-Penguin is higher than that of cipher-Lena, but the min-entropy is just the opposite. According to attacking method, cipher-Penguin is easier to break. From such fact, we think min-entropy is better than Shannon entropy in security analysis. In addition, Table 4 shows that the min-entropy of our scheme is higher than that of the scheme in literature [11]. Thus, our scheme is more secure.

### 5.8. Speed Analysis

Apart from the security aspect, running speed is another important factor in measuring image encryption scheme. Due to the difference of computer configurations and code optimization ways, running speed cannot be compared directly [13]. Therefore, time complexity analysis is used. Our algorithm includes two stages. They are forward and backward encryption. Time complexity of each stage is *O*(*LN*), here, *L*denotes feedback maximum length. Therefore, the whole algorithm time complexity is *O*(2*LN*). The time complexity of paper [22] is *O*(*L* + 3*N* + *kN*log⁡(*N*)). Thus, our algorithm is effective in speed.

Additively, in our scheme, several measures are taken to improve running speed. The first is disturbing chaotic stream by cipher-text which can effectively avoid wasting chaotic number and hence accelerate key generation. The second is only two rounds which are necessary to implement high sensitivity of cipher-text to any position plaintext. All these measures can help to accelerate the running speed.

## 6. Conclusion

In this paper, we present a new image encryption algorithm based on one time running-key. Using cipher-text to disturb chaotic stream not only helps to minimize digital chaotic degradation, hence reducing the influence of degradation on security of an encryption system, but also implements one time running key and improves the encryption efficiency. Also, introducing random feedback mechanism further enhances the security of our scheme. In addition, min-entropy, which more exactly measures the ability of resisting statistical attack, is first proposed to replace Shannon entropy. The last feature is chaotic map and transformation function which can be chosen according to user requirement and possessing strong flexibility and expandability. In the future, parallel implementation of image encryption scheme will be investigated.

## Figures and Tables

**Table 1:** Correlation of adjacent pixels.

	Correlation		
	Horizontal	Vertical	Diagonal
Lena image	0.9432	0.9730	0.8887
Rand image	0.0122	0.0161	−0.0141
Lena cipher image	−0.0278	−0.0065	−0.0074

**Table 2:** Sensitivity analysis.

Test items		Alter	Result
Change only one bit	NPCR	First pixel	0.9966
		Last pixel	0.9962
	UACI	First pixel	0.3352
		Last pixel	0.3357
	Avalanche effect		0.5004

**Table 3:** Sensitivity test.

Parameter				Initial value		
Δ	Δ*r*_1_	Δ*r*_2_	Δ*r*_3_	Δ*x*_1_	Δ*x*_2_	Δ*x*_3_

Difference	10^−14^	10^−14^	10^−14^	10^−14^	10^−14^	10^−14^
Correlation	−0.0042	−0.0080	0.0041	−0.0022	0.0092	−0.0030

**Table 4:** Information entropy.

	Shannon entropy	Min-entropy
Lena image		
Plain-image	7.4420	6.5304
Cipher [11]	7.9894	7.6469
Our cipher	**7.9895**	**7.7857**
Penguin image		
Plain-image	3.1176	1.4634
Cipher [11]	7.9897	7.7568
Our cipher	**7.9904**	**7.7664**
